# Venous Insufficiency: Endovascular and Surgical Treatment

**DOI:** 10.1007/s11886-024-02155-x

**Published:** 2025-03-06

**Authors:** Abdullah Khan, Ahmed A. Sorour, George E. Anton, Sean P. Lyden, Lee Kirksey

**Affiliations:** Department of Vascular Surgery, Heart, Vascular & Thoracic Institute, Cleveland Clinic, 9500 Euclid Ave, F30, Cleveland, OH 44195 USA

**Keywords:** Chronic venous insufficiency, Radiofrequency Ablation, Endovenous Laser Ablation, Mechanochemical ablation, Sclerotherapy, High Ligation and Stripping

## Abstract

**Purpose of review:**

Venous insufficiency (VI) is a is a common and debilitating disease that can present with a wide range of manifestations ranging from telangiectasias to venous ulceration. The chapter explores various endovascular and open-surgical modalities used for VI, their technique, patient selection, outcomes, complications, and comparison with other modalities.

**Recent findings:**

The use of non-thermal and non-tumescent ablation techniques are found to have a better quality of life scores; however, the primary closure rates are inferior to thermal ablation techniques.

**Summary:**

A wide range of treatment modalities are available, ranging from conservative management, endovascular techniques and open-surgical options. Each intervention has its unique benefits, limitations, and potential complications. The management approach for VI is not one-fits-all, and must be meticulously tailored to each patient, carefully considering their disease severity, anatomy, quality of life, and expectations, for an effective treatment.

## Introduction

Venous Insufficiency (VI) is often overlooked by healthcare providers despite its very high prevalence in the United States, especially when compared to other vascular diseases [1]. The condition causes disruption in deep/superficial venous flow, caused by venous valve reflux, venous flow obstruction, or both, which leads to increased venous pressure [2]. Venous Hypertension presents with a myriad of symptoms, including telangiectasia, varicose veins, leg pain, pruritus, limb heaviness, edema, skin discoloration, corona phlebectatica, and venous ulcers [3, 4]. Due to a wide array of symptoms related to chronic venous diseases (CVD), a standardized Clinical-Etiology-Anatomy-Pathophysiology (CEAP) classification system was introduced in 1996, with a recent update in 2020. It provides a reproducible and dependable classification system for complex manifestations of CVD [5, 6]. The revised Venous Clinical Severity Score (VCSS) is another evaluation system used routinely, which caters to changes over time and helps document the response to treatment [7]. Gloviczki et al. recommend Doppler Ultrasound (DU) as a noninvasive diagnostic test of choice for CVD [7]. The symptoms associated with VI cause significant patient disability, impaired function, and reduced quality of life. For that reason, quantifying the improvement of patient-reported outcome variables derived from successful management and intervention is very important.

The management of CVD is quite intricate, with various options of conservative, pharmacological therapy, open surgical, and endovascular options available. Patients with > 500ms of retrograde venous flow/axial reflux and/or symptomatic patients are considered for management [7]. Gloviczki et al. recommend compression therapy in symptomatic patients if the ambulatory status and/or underlying medical conditions warrant a conservative approach, but if patients are candidates for intervention, superficial venous intervention is suggested over long-term compression therapy [8]. Pharmacological therapy can be considered for symptomatic patients with varicose veins who are not candidates for intervention or those waiting for intervention or have symptoms after intervention, but the products available are not Food and Drug Administration (FDA) approved [9, 10]. Open surgical techniques and endovascular options are recommended as viable treatment modalities per Society for Vascular Surgery (SVS) and American Venous Forum (AVF) guidelines, with a preference for Endovascular procedures due to improved periprocedural and postprocedural outcomes [7, 11, 12].

## Pre-operative planning

A thorough history and a detailed physical examination remain a cornerstone for managing VI. However, various imaging modalities can be employed to assess etiology and clearly define the anatomy. Venous anatomy is variable in patients with VI, especially in patients who have had prior procedures. DU, which combines B-mode imaging and spectral doppler waveform with provocative maneuvers to identify venous obstruction and reflux, is the gold standard diagnostic imaging modality [7, 13]. Distal segments and reflux are evaluated by manual compression or cuff inflation-deflation technique [14]. DU provides anatomic mapping of the reflux disease in the deep and superficial venous systems and perforator veins. The reflux time of > 0.5s and 1.0s for superficial and deep veins is considered diagnostic for reflux with a direct correlation between reflux time and disease severity [7, 15].

Air plethysmography is another vital technique to identify reflux and obstruction in the venous system. It can be clinically helpful in patients with non-diagnostic DU, especially in class C3-C6 [1, 16]. Similarly, computed tomography (CT) and magnetic resonance (MR) venography can help delineate complex venous anatomy and surrounding structures that may cause venous obstruction and evaluate more proximal veins like the iliofemoral vein segment [17, 18]. Contrast venography, though invasive, can help differentiate primary and secondary disease and is most diagnostic for reflux in the common femoral and saphenofemoral junction [1]. Intravascular ultrasound is a newer technique and is superior to venography in determining the severity and significance of stenosis and intraluminal anatomy [19]. The imaging modality must be selected according to the patient's presentation and comorbidities. The best opportunity to understand the anatomy is to bond with the registered vascular technologist by reviewing important findings live, sharing thoughts, and knowing that the endpoint of the study is when you can accurately formulate a plan of action and not simply complete a standard protocol.

## Endovascular Techniques

Endovascular techniques can be divided into Thermal ablation and non-thermal, non-tumescent ablation. Thermal ablation techniques involve the use of either radiofrequency or lasers, whereas non-thermal and non-tumescent ablation techniques include chemical, mechanochemical, and adhesive subtypes [20]. The main goal of treating VI is the closure of superficial veins with reflux, starting at the most proximal point and attaining good cosmetic results. Endovascular ablation techniques are recommended over surgical options for patients with symptomatic varicose veins and axial reflux in great saphenous vein (GSV) and small saphenous vein (SSV) [12]. Kheirelseid et al. reported that Radiofrequency and endovenous laser ablation are as effective as surgery for treating saphenous vein insufficiency at 5 years [21]. Endovascular procedures are associated with decreased risk of post-procedure pain, need for analgesia, adverse effects, and improved time for return to normal activities, and SF-36 quality of life patient scoring at 6 weeks. [22–24]. Table 1 presents a brief comparison of the endovascular procedures.

**Table 1:** Comparison of mechanism, contraindications, complications, preference, and need for tumescent anesthesia between different types of endovascular techniques, i-e RFA, EVLA, Adhesive closure, MOCA, and Sclerotherapy

Technique	Description	Mechanism	Contraindications (absolute or relative)	Complications	Preferred for/if	Tumescent anesthesia needed
Radiofrequency Ablation (RFA)	Thermal ablation using Radiofrequency energy to seal off refluxing veins	Thermal energy to vein wall causes collapse and closure	SVT, DVT, pregnancy aneurysmal veins, bleeding/clotting disorders, ABI < 0.9,	Paresthesia, hyperpigmentation, hematoma, ecchymosis, phlebitis	- Decreased post-op pain, ecchymosis and swelling over EVLA	Yes
Endovenous Laser Ablation (EVLA)	Thermal ablation using Laser energy to seal off refluxing veins	Laser energy produces heat for Fibrosis and vein closure	DVT, pregnancy, veins < 1cm to skin, aneurysmal veins	Perforation, EHIT paresthesia, ecchymosis, phlebitis	- Tributaries are involved, improved venous ulcer resolution in Stages C5-C6 disease, over RFA	Yes
Adhesive Closure (Cyanoacrylate)	Uses medical adhesive to seal off refluxing veins	Adhesive-induced vein thrombosis	Hypersensitivity, acute infections, previous reaction to cyanoacrylates	Hypersensitivity, skin reactions	- Higher short and long-term ablation rate compared to RFA and to avoid nerve injury	No
Mechanochemical Ablation (MOCA)	Direct mechanical damage and sclerosing agent to seal off refluxing veins	Mechanical damage causes vein to spasm + sclerosing agent further thrombosis	Less effective for larger veins or complex cases	Lower closure rates than thermal methods, less common issues	- Improved venous ulcers resolution compared to thermal ablation	No
Sclerotherapy	Sclerosants injected into thrombose and seal off refluxing veins	Chemical-induced fibrosis and thrombosis of refluxing vein segment	Cellulitis, thrombophlebitis, pregnancy, bedridden patients	Hyperpigmentation, telangiectatic matting, pain, urticaria	- Smaller diameter veins (< 5mm), varicose tributaries are present, cosmetic treatment - Used concomitantly with other techniques	No

### Thermal Ablation

#### Radiofrequency Ablation (RFA)

**Fig. 1:**
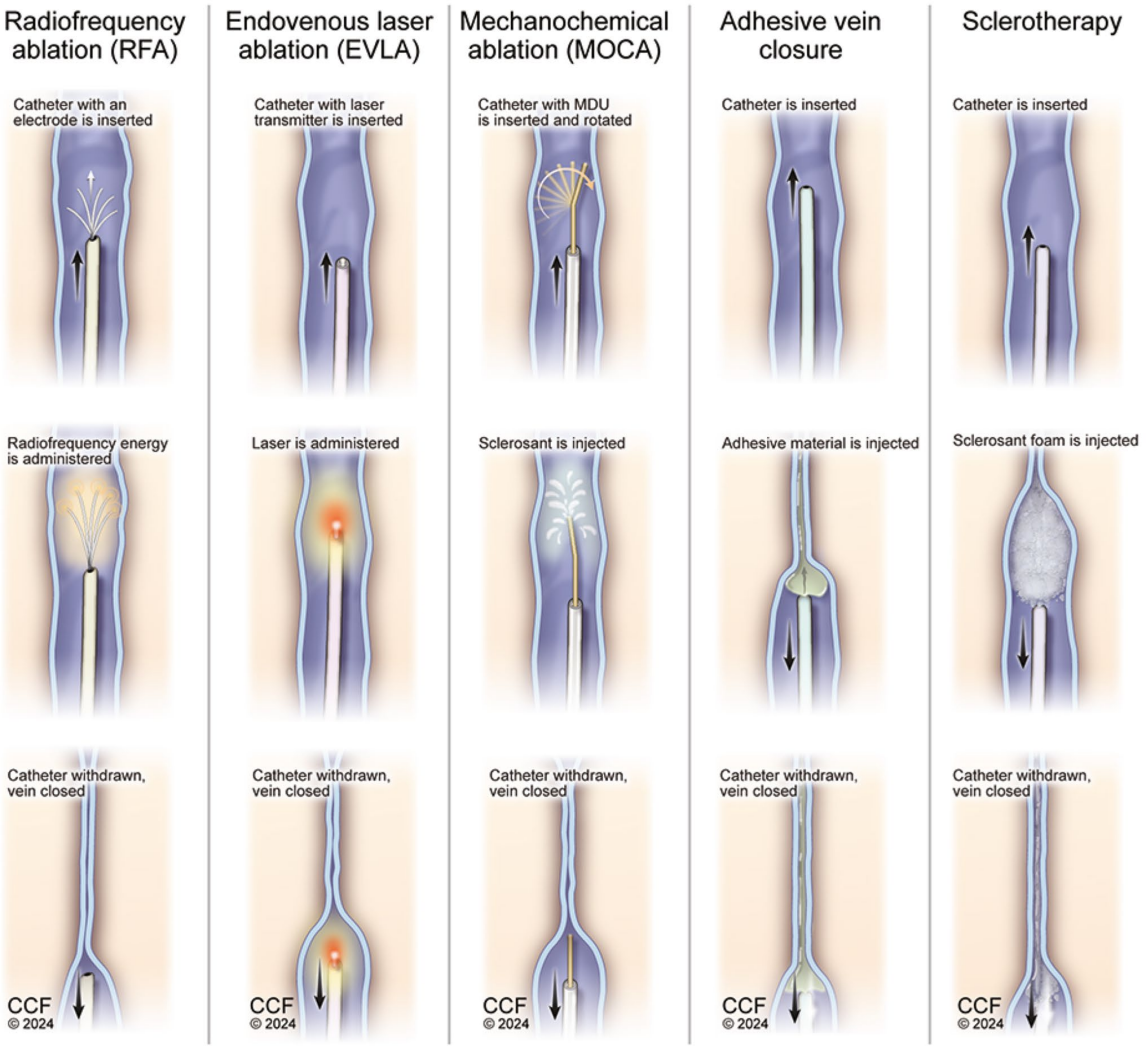
Differences in the mechanism of action for various endovascular techniques, i-e RFA, EVLA, Adhesive closure, MOCA, and sclerotherapy

RFA is a minimally invasive technique routinely performed in an outpatient setting, which closes the refluxing vein by providing controlled and targeted thermal energy [Fig. 1]. It is performed where veins are deep to the saphenous fascia to minimize the risk of thermal injury. The electrode of the RFA catheter transmits radiofrequency energy when it encounters the vein, which causes destruction of the endothelium, contraction of the vein wall, and thrombus formation [21, 25, 26].

The skin is anesthetized with local lidocaine, and access to the vein is achieved percutaneously using ultrasound guidance at a point on the leg distal to the region of venous reflux. Tumescent anesthesia is delivered in perivenous tissue with the goal of covering the vein with tumescence in the saphenous fascia to ensure adequate anesthesia and to form a barrier for heat transfer to deep veins and skin. The RFA catheter is advanced through the sheath. The catheter tip is visualized distal to the epigastric venous complex. The patient is placed into the Trendelenburg position to facilitate vein collapse, and treatment cycles are given to the affected areas [24, 27].

The are no absolute contraindicated for RFA use, but the relative contraindications include superficial vein diameter of less than two mm, superficial venous thrombosis (SVT), deep venous thrombosis (DVT), venous aneurysm, pregnancy, known malignancy and uncorrectable bleeding or clotting disorders, and an ankle-brachial index of less than 0.9 [20, 25]. Post-procedure Compression therapy is recommended for one week after undergoing thermal ablation [7, 11]. Some studies report no difference in patient-reported outcomes between patients who underwent compression therapy and those who did not [28].

Commonly reported complications of RFA are paresthesia, phlebitis, erythema, ecchymosis, hematoma, endothermal heat-induced thrombosis (EHIT), and hyperpigmentation [29]. Proebstle et al., with a three-year follow-up for RFA, reported a very low incidence of complications, with the majority resolving in the first week. Complications beyond the first week only included Pigmentation and Paresthesia [30]. Shepherd et al. compared procedural pain with RFA and EVLA and reported RFA to be associated with lower postoperative pain scores at three days (14.5 vs. 25.8) and ten days (13 vs. 23.3) [31].

Ablation rates within the treated segments were observed to be 96.3%, 94.5%, and 92.6% at 12, 24, and 36 months [30]. Ablation rates of 92.6% and 91.9% are observed at three- and five-year follow-ups. Pain improvement was reported as patients free of pain were 41% before intervention and 98% at three-year follow-up. Improvement in VCSS was noted from an average of 3.9 before treatment to 0.9 and 1.3 at three and five years, respectively [30, 32].

#### Endovenous Laser Ablation (EVLA)

EVLA is also a minimally invasive technique with many procedural similarities to RFA. EVLA uses different laser wavelengths and types of fibers to deliver laser energy. [Fig. 1] Laser energy is focused on a target area which causes fibrosis and occlusion of the reflux segment. Studies have shown no significant difference in the efficacy of laser wavelengths, but higher wavelengths are associated with a lower relative incidence of pain reported during the procedure and less periprocedural ecchymosis [33, 34]. In comparison with bare-tip fiber, radial, and jacket-tip fibers are associated with less pain and bruising. Radial and jacket-tip fibers avoid direct contact with the vein, preventing perforation and, hence, pain and ecchymosis [35, 36].

The catheter is advanced using the same technique in RFA; the tip of the fiber is exposed 2cm past the sheath’s end. The position of the tip is confirmed with ultrasound; tumescence is administered, and the Laser is commonly delivered at 40 J/cm and 80 J/cm settings. Laser fiber and sheath are pulled back together 1cm every 3–5 s [22]. The mean energy delivered for successful treatment was reported to be 63.4 ± 26.6 J/cm [37].

Discharge, follow-up, and indications of EVLA and RFA are similar [7, 11]. EVLA is contraindicated in patients with a history of DVT and who are currently pregnant. Whereas, Chronic or recurrent phlebitis, severe tortuosity, target veins < 1cm from skin, and aneurysmal target GSV (> 2.5cm diameter) are relative contraindications to EVLA [20].

Complications associated with EVLA are low in incidence, but it may lead to vessel perforation, endothermal heat-induced thrombosis (EHIT), phlebitis, ecchymosis, paresthesia, skin necrosis, and pain. Though uncommon, EHIT is crucial to classify and manage due to its potential to lead to pulmonary embolism. (Table 2) A meta-analysis by Healy et al. compared complications between EVLA and RFA, finding that EVLA had a lower proportion of DVT (0.002 vs. 0.005) and EHIT (0.01 vs. 0.012), while both treatments had a similar incidence of PE (0.001) [38].

**Table 2:** Endothermal heat-induced thrombosis (EHIT) classification by American Venous Forum (AVF), and treatment strategies for EHIT after ablation of great saphenous vein (GSV) and small saphenous vein (SSV), as recommended by the Society for Vascular Surgery (SVS) and AVF. (74].

Class	Definition	Treatment	Evidence
1a	Thrombus distal to superficial epigastric vein without propagation into the deep vein	No treatment or surveillance	2C
1b	Thrombus proximal to superficial epigastric vein without propagation into the deep vein	No treatment or surveillance	2C
2	Thrombus propagation into the adjacent deep vein but comprising < 50% of the deep vein lumen	**Low Risk:** No treatment, only weekly surveillance until thrombus resolution **High-risk:** Antiplatelets vs. prophylactic or therapeutic anticoagulants with weekly surveillance until thrombus resolution, may be considered	2C
3	Thrombus propagation into the adjacent deep vein but comprising > 50% of the deep vein lumen	Therapeutic anticoagulation with weekly surveillance until thrombus retraction or resolution to the GSV or SSV junction	1B
4	Occlusive deep vein thrombus contiguous with the treated superficial vein	Managed as an occlusive DVT with an individualized approach considering the risks and benefits to the patient following CHEST Guidelines. (73].	1A

The outcomes of EVLA for 810-nm laser reported an ablation rate of 98.2% at two years, whereas for 1320-nm, 940-nm(15W), and 940-nm (30W) lasers showed ablation rates at three months of 97%, 90.3%, and 100% respectively [33, 39, 40]. Desmyttère et al. reported an ablation rate of 98% at one week and 97.1% at four years for a 980-nm laser [41].

### Non-thermal, non-Tumescent Ablation Techniques

#### Mechanochemical Ablation (MOCA)

Thermal ablation techniques have several disadvantages, i.e., thermal damage to nerves causing paresthesia, skin burns, and the need to use tumescent anesthesia. MOCA overcomes these disadvantages by using a catheter with a rotating motor drive unit (MDU), which causes mechanical damage to the endothelium, causing it to spasm. Along with causing mechanical spasms, the MOCA technique uses a sclerosing agent, which leads to further endothelial damage and closure of the refluxing vein [Fig. 1] [22, 42].

Witte et al. performed a systematic review of 1521 veins and reported primary closure rates of 92%, 91%, 91%, and 87% at six months, one-year, two years, and three years follow-ups, respectively [43]. The LAMA trial showed occlusion rates of 91% and 77% with EVLA and MOCA, respectively, and reported significant improvement in VCSS and Aberdeen varicose vein questionnaire (AVVQ) scores [44]. MOCA demonstrated improved venous ulcer healing in comparison with thermal ablation techniques [45]. A recent meta-analysis has produced some skepticism regarding closure rates and improvement in VCCS scores with MOCA. Results demonstrated RFA, EVLA, and cyanoacrylate ablation with increased odds of primary closure compared to MOCA and similar trends in the improvement of VCSS scores [46].

#### Adhesive Closure

This technique delivers n-butyl cyanoacrylate adhesive material to the target area leading to vein closure without the aid of thermal energy or tumescent anesthesia. When delivered in the refluxing vein, it polymerizes and thrombosis the vein segment and stays as a permanent implant [Fig. 1] [22, 47].

Almeida et al. reported the first human use of cyanoacrylate glue closure (CAC) with a closure rate of 92% at one and two-year follow-ups and improvement in VCSS scores from a mean baseline of 6.1 to 1.5 and 2.7 at one and two-year follow-ups [47, 48]. VeClose Trial compared the efficacy of RFA vs CAC for incompetent GSV; the trial shows closure rates of 95.3% and 91.4% in the CAC group, whereas 94% and 85.2% in the RFA group at two years and five years, respectively. Symptoms and quality of life improved similarly in both groups [49, 50].

Contraindicated in patients with thrombophlebitis migrans, acute superficial thrombophlebitis, Previous hypersensitivity reaction to cyanoacrylates, and acute sepsis. Common complications associated with CAC are hypersensitivity reactions, hyperpigmentation, acute infection, residual bulkiness of the treated vein, and thromboembolic events. Hypersensitivity was the most common complication, which occurred in 5.6–6.3% of the patients [51, 52].

#### Sclerotherapy

This technique involves a chemical agent used to cause endothelial damage leading to fibrosis and closure of the refluxing vein and can be used to treat many vein types and sizes [Fig. 1]. The choice of method to deliver the sclerosing agent depends on the size and type of the target vein [53]. Liquid sclerotherapy is used for smaller veins, whereas foam sclerotherapy is preferred for larger vein diameters. Various sclerosing agents are available and are categorized based on their mechanism, i.e., osmotic, alcohol, and detergent [54, 55]. Catheter directed sclerotherapy can also be used with sclerosant injected using catheters under DU guidance with comparable ablation rates and reduced complications [56].

Sclerotherapy is contraindicated in patients with cellulitis, acute respiratory disease, phlebitis migrans, superficial thrombophlebitis, pregnancy, bedridden status, and hyperthyroidism [53]. Common complications include hyperpigmentation, telangiectatic matting, pain, and urticaria. Hyperpigmentation is the most common complication (10–30%) and resolves in 99% of patients at one-year intervals [57, 58]. DVT may occur in 1–3% patients, and rarely may lead to anaphylaxis, and thrombophlebitis [53, 57, 58]. Although rare, it may cause a stroke through paradoxical embolism, especially in patients with patent foramen ovale [59].

The primary closure rates varied with the agent and technique used. Kölbel et al. indicated closure rates of 90.2% and 92.2% for STS liquid sclerotherapy and foam sclerotherapy, respectively [60]. STS Foam sclerotherapy with the Tessari method reported a 93.3% primary closure rate [61]. Polidocanol foam sclerotherapy reported a 79.8% primary closure rate [62].

## Surgical Techniques

### High Ligation and Stripping (HL&S)

This surgical technique involves pre-operative DUS marking of saphenofemoral junction (SFJ) for small and precise incision; after the skin incision, the main trunk of GSV is identified, and small branches of the femoral artery are ligated for better exposure of SFJ. High ligation of GSV is done close to the femoral vein. If high ligation alone is planned, the exposed GSV segment of 5-10cm is removed. If stripping is performed in conjunction, only the incompetent segment is excised, leaving the normal distal vein. A stripper is passed distally, with a second small incision at the level of the knee; all tributaries are avulsed, caudal GSV divided and ligated, and a vein stripper is drawn from the groin incision. The procedure is performed under DUS guidance, and tumescent anesthesia is provided [63]. The leg is elevated before and during a procedure to reduce bleeding and ecchymosis. The most common complications are hematoma, cellulitis, abscess, numbness, tingling, DVT, and pseudo-thrombophlebitis [64]. At a five-year follow-up, EVLA was associated with a lower recurrence rate (20.9%) compared to surgery (31.3%). EVLA also elucidated clear improvement in VCSS scores [65]. Pain scores, six of eight SF-36 domains, and quality-adjusted life years (QALYs) were significantly different between EVLA and surgery, favoring EVLA and leading to early return to work and normal activity with EVLA (4 days vs. 14 days with surgery) [66]. These factors compounded the SVS and AVF to recommend endovascular ablation techniques over HL&S [11].

### Ambulatory Phlebectomy

This technique involves < 2mm horizontal skin incisions with 11 blade along Langer’s skin tension lines for better cosmesis, the vein is retrieved through the incision, and small varicosities are hooked and avulsed, whereas larger varicosities are pulled through the incision for an avulsion. Skin is usually closed with sterile strips, and sutures are rarely needed [67]. Transilluminated powered phlebectomy involves transillumination of the veins, dissection with tumescent anesthesia, and powered phlebectomy. This technique decreases the number of incisions and operative time with comparable patient-reported outcomes to conventional phlebectomy [68].

### Saphenous Sparing Operations

Labropoulos et al. hypothesized that the cause of varicose veins initiated in the distal superficial venous segment, and thus removing peripheral venous varicosities is deemed necessary while sparing the saphenous vein [69]. Ambulatory selective varicose vein ablation under local anesthesia (ASVAL) and 'cure conservatrice et hémodynamique de l'insuffisance veineuse en ambulatoire' (CHIVA) are successful techniques under this guiding theory.

CHIVA technique is usually a two-step procedure that treats the point when reflux starts and collaterals that might contribute to reflux while preserving the re-entry point to the deep venous system and sparing the saphenous vein. CHIVA has little or no difference in clinical recurrence rates when compared to HL&S, RFA, and EVLA but causes a slightly reduced risk for nerve injury and hematoma formation in comparison with HL&S [70]. Patients with CHIVA had lower post-operative pain scores in comparison to EVLA [71].

ASVAL involves the removal of varicosities through multiple stab avulsions, which is a modification of the old Madelung method. It involves peripheral varicosities while sparing SFJ and saphenous vein. ASVAL is associated with the absence of saphenous reflux in 64.4%, varicose recurrence in 68.8%, functional improvement in 69.9%, and esthetic improvement in 65.7% of the cases at ten years follow-up [72].

### Multidisciplinary Collaboration

Venous treatments have evolved to include surgical and non-surgical treatments involving vascular surgery, interventional radiology, cardiology, vascular medicine, and other fields. Collaboration among specialists offers the best opportunity to match technology with a patient’s anatomy in an unbiased fashion. A multispecialty collaborative setting will offer cognitive diversity to fully understand the complexities of venous disease and offer the most effective treatment strategies for each patient’s unique presentation. This approach will minimize confirmation bias and resultant plan continuation bias that may interfere with sound clinical judgment and decision-making.

## Conclusions

In managing venous insufficiency, the selection of surgical and endovascular techniques must be individualized, accounting for the patient's anatomy, clinical presentation, and CEAP classification while considering the patient’s expectations. Each intervention carries specific benefits, limitations, and potential complications. Conservative management may be appropriate initially for certain patients, but when intervention is indicated, current SVS and AVF guidelines recommend endovascular procedures over HL&S, if expertise is available. It is also recommended that Endovascular techniques be accompanied by compression therapy for at least one week. In certain cases, adjunctive procedures such as phlebectomy or sclerotherapy may be incorporated into the treatment regimen. Additionally, for patients with early-stage symptomatic varicose veins, techniques like CHIVA or ASVAL may be considered when preserving the saphenous veins is a priority. Ultimately, the management approach must be meticulously tailored to each patient, with careful consideration given to the appropriate technique along with a potential need for multiple interventions. The management for VI is not one-fits-all, and the techniques need to be personalized and sometimes overlap with other interventions for effective treatment.

## Key References

- Gloviczki P, Lawrence PF, Wasan SM, et al. The 2022 Society for Vascular Surgery, American Venous Forum, and American Vein and Lymphatic Society clinical practice guidelines for the management of varicose veins of the lower extremities. Part I. Duplex Scanning and Treatment of Superficial Truncal Reflux: Endorsed by the Society for Vascular Medicine and the International Union of Phlebology. J Vasc Surg Venous Lymphat Disord. 2023;11(2):231–261.e6. 10.1016/j.jvsv.2022.09.004
  - ⚬ This study provides the guidelines for diagnosing chronic venous insufficiency and management of superficial truncal reflux.
- Gloviczki P, Lawrence PF, Wasan SM, et al. The 2023 Society for Vascular Surgery, American Venous Forum, and American Vein and Lymphatic Society clinical practice guidelines for the management of varicose veins of the lower extremities. Part II: Endorsed by the Society of Interventional Radiology and the Society for Vascular Medicine. J Vasc Surg Venous Lymphat Disord. 2024;12(1):101,670. 10.1016/j.jvsv.2023.08.011
  - ⚬ This study provides the guidelines for the management of varicose veins and chronic venous insufficiency.
- Farah MH, Nayfeh T, Urtecho M, et al. A systematic review supporting the Society for Vascular Surgery, the American Venous Forum, and the American Vein and Lymphatic Society guidelines on the management of varicose veins. J Vasc Surg Venous Lymphat Disord. 2022;10(5):1155–1171. 10.1016/j.jvsv.2021.08.011
  - ⚬ This systematic review supports the use of duplex for evaluating patients with varicose veins and found thermal ablation with lower risk of recurrent incompetence than non-thermal ablation.

## Data Availability

No datasets were generated or analysed during the current study.
